# Systemic and Cardiac Depletion of M2 Macrophage through CSF-1R Signaling Inhibition Alters Cardiac Function Post Myocardial Infarction

**DOI:** 10.1371/journal.pone.0137515

**Published:** 2015-09-25

**Authors:** Anne-Laure Leblond, Kerstin Klinkert, Kenneth Martin, Elizebeth C. Turner, Arun H. Kumar, Tara Browne, Noel M. Caplice

**Affiliations:** Centre for Research in Vascular Biology (CRVB), Biosciences Institute, University College Cork, College Road, Cork, Ireland; Albert Einstein College of Medicine, UNITED STATES

## Abstract

The heart hosts tissue resident macrophages which are capable of modulating cardiac inflammation and function by multiple mechanisms. At present, the consequences of phenotypic diversity in macrophages in the heart are incompletely understood. The contribution of cardiac M2-polarized macrophages to the resolution of inflammation and repair response following myocardial infarction remains to be fully defined. In this study, the role of M2 macrophages was investigated utilising a specific CSF-1 receptor signalling inhibition strategy to achieve their depletion. In mice, oral administration of GW2580, a CSF-1R kinase inhibitor, induced significant decreases in Gr1^lo^ and F4/80^hi^ monocyte populations in the circulation and the spleen. GW2580 administration also induced a significant depletion of M2 macrophages in the heart after 1 week treatment as well as a reduction of cardiac arginase1 and CD206 gene expression indicative of M2 macrophage activity. In a murine myocardial infarction model, reduced M2 macrophage content was associated with increased M1-related gene expression (IL-6 and IL-1β), and decreased M2-related gene expression (Arginase1 and CD206) in the heart of GW2580-treated animals versus vehicle-treated controls. M2 depletion was also associated with a loss in left ventricular contractile function, infarct enlargement, decreased collagen staining and increased inflammatory cell infiltration into the infarct zone, specifically neutrophils and M1 macrophages. Taken together, these data indicate that CSF-1R signalling is critical for maintaining cardiac tissue resident M2-polarized macrophage population, which is required for the resolution of inflammation post myocardial infarction and, in turn, for preservation of ventricular function.

## Introduction

Cardiac tissue-resident macrophages, derived from circulating monocytes, exhibit an alternatively activated or M2 phenotype expressing cytoprotective factors and immune repressors such as IL-10 [1]. Following the onset of MI, a biphasic monocyte and macrophage response ensues [2] consisting of an early inflammatory phase of classically activated M1 macrophages that release proinflammatory cytokines such as TNF- α, IL-1β, reactive oxidative species, as well as extracellular matrix modifying MMPs. This phagocytic response is followed by a presumed reparative phase involving M2 macrophage activity in which inflammation is thought to be dampened along with stimulation of tissue repair, matrix production and angiogenesis [3]. To date, no study has altered the macrophage phenotypic balance between M1 and M2 to definitively test the role of M2 phenotype cells in repair post MI.

Macrophage maturation from monocyte precursors is mainly driven by signaling through colony stimulating factor receptor 1 (CSF-1R) and its ligand CSF-1, which stimulates proliferation, differentiation, chemotaxis towards CSF-1 and survival of monocytes and macrophages. Binding of CSF-1 leads to autophosphorylation of receptor subunits and regulation of different downstream molecules, thus leading to the pleiotropic effect of CFS-1 signalling. CSF-1 promotes furthermore the differentiation towards M2 anti-inflammatory macrophages, which can be inhibited by TNF-α. TNF-α is released by neutrophils and M1 macrophages promoting a pro-inflammatory environment and can thus modulate macrophage plasticity.

CSF-1 signalling, and its inhibition *in vivo*, using a small molecule inhibitor of cFMS kinase activity (GW2580) has been investigated in animal models of inflammatory disease [4].

However, in the models of acute kidney injury, GW2580 treatment exacerbated the renal injury and fibrosis through a significant decrease in resident macrophages and a marked increase in M1 markers [5]. Moreover it has been shown that local overexpression of the ligand CSF-1 may be beneficial for cardiac repair in a model of heart failure in rats [6].

We hypothesized that M2 macrophage depletion in the heart will affect the cardiac repair response post infarction. Accordingly, as M2 macrophage polarity is driven in large part by of CSF-1 [7,8] we adopted an *in vivo* CSF1 inhibition strategy, using a small molecule inhibitor.

## Methods

### Animals

All procedures were approved by University College Cork Animal Experimentation Ethics Committee (AEEC) in addition to being licensed by the Department of Health and Children in Ireland. Procedures were performed under anesthesia induced by ketamine (90mg/kg), xylazine (10mg/kg) and urethane (1.25mg/kg) and analgesia by i.p. delivery of carprofen (5 mg/Kg). Adult male C57BL6/J and MAFIA mice (C57BL/6J-Tg(Csf1r-GFP, NGFR/FKBP12)2Bck/J) [9] were obtained from Harlan Laboratory (UK) and Jackson Laboratory with MTA from Ariad Pharmaceuticals Inc (Cambridge, MA), respectively.

### In vivo CSF-1R inhibition by GW2580 administration

GW2580 (Biorbyt, UK; CSF1 receptor kinase inhibitor [10]) diluted in a vehicle solution (0.5% hydroxy propyl methylcellulose and 0.1% Tween 80) or vehicle solution alone was administered once daily at 160 mg/kg per dose by oral gavage using stainless steel feeding tubes (Instech Solomon, US). Previous investigations have established that GW2580 administered at 160 mg/kg remains at an optimal therapeutic blood plasma concentration greater than 1μm for 24h [10] [11]. Male, 8 weeks-old C57BL6/J and MAFIA mice were under GW2580 or vehicle treatment for one week prior and two weeks following infarction.

### Flow cytometry

Cells from peripheral blood, heart and spleen were collected for cytometric analysis using a BD FACSAria II cytometer (Becton Dickinson, Erembodegem, Belgium). Blood, heart and splenic cells were stained with antibodies against Ly6G to determine neutrophils (BD Pharmingen), and CD206 (BioLegend), Gr1 (Serotec) and F4/80 (Caltag) to determine monocyte and macrophage subtypes. Reported percentages were gated on GFP^+^ cells (CSF+1^+^ cells in MAFIA mice). M2 macrophages were identified as CD206^+^ cells. Total macrophage population (M1 and M2) was defined as F4/80^high^. Total M1 population (monocytes and macrophages) were Gr1^+^ cells. Total monocyte population (M1 and M2) was defined as F4/80^low^. Appropriate isotype controls, as well as unstained samples, were used as negative controls (S1 Fig).

### Myocardial Infarction

C57BL6/J and MAFIA animals underwent a myocardial infarction surgery by permanent left anterior descending (LAD) artery. Mice were anesthetized with ketamine (90mg/kg), xylazine (10mg/kg) and urethane (1.25mg/kg). The animals were intubated and ventilated with a Harvard Rodent ventilator. Body temperature was monitored and regulated at 37°C throughout the procedure. Left thoracotomy between the 3^rd^ and the 4^th^ intercostal space was performed to expose the left ventricle. After removing the pericardium, the LAD artery was ligated with an 8–0 suture placed 0.3mm below the tip of the left atrium. Occlusion was confirmed by observation of left ventricular pallor immediately post ligation. Five minutes after LAD artery ligation, 360 000 fluorescent microspheres (10 μm size, excitation/emission wavelength 580/605 nm; Fluospheres, Invitrogen) were slowly injected in a volume of 100 μl into the left ventricle, and distributed to myocardial areas with intact perfusion. The chest was closed and the animal kept under mechanical ventilation until spontaneous breathing occurred and recovered on a heating pad.

### Hemodynamic assessment using conductance catheters

Pressure-volume loop experiments were carried out 2 weeks post MI and GW2580 treatment. 1.4F Millar pressure volume catheter (AD Instruments, UK) composed of four conductance electrodes was used to record the pressure volume data. Mice were anesthetized by intraperitoneal administration of a mixture of ketamine (90mg/kg), xylazine (10 mg/kg) and urethane (1.25mg/kg). The animals were placed in the supine position under a dissecting microscope, and a vertical midline cervical incision was made. The right carotid artery and external jugular vein were exposed via the same midline incision. The 1.4F Millar pressure volume catheter was advanced via the right carotid artery into the ascending aorta and then inserted into the left ventricule (LV). The LV pressure-volume data were recorded as a series of pressure-volume loops (about 50 loops) and analysed with PVAN Ultra 1.0 software (Millar Instruments Inc, USA). Parallel volumes for each mouse and correction for loading conditions was calibrated by the injection of 150μl of hypertonic saline into the tail vein.

### Tissue preparation

One week following GW2580 daily administration, MAFIA mice were euthanized by overdose of urethane. Cells from 1) peripheral blood, 2) heart, and 3) spleen were collected for further cytometry staining as follows; 1) Peripheral blood was drawn by facial vein puncture with 0.5M EDTA. Red blood cells were lysed by ACK lysis buffer. 2) The chest was opened and the heart was perfused with 3mls of chilled PBS. The heart was then excised, washed in PBS and minced with sharp scissors. To obtain a single cell solution the minced heart was digested in a collagenase type II (0.15mg/ml) /pancreatin (0.52mg/ml) solution in ADS buffer at 37°C in the dark while shaking. After twenty minutes, the cell suspension was neutralized with 0.5M EDTA and the remaining tissue was digested for a further 20 mins. The cell suspensions were pooled, pushed through a 40μm cell strainer and centrifuged for 5min at 600xg. 3) The spleen was excised, washed in PBS and perfused with collagenase type II (0.15mg/ml). After 5 min of incubation at RT, the spleen was pushed through a 40μm cell strainer and centrifuged for 5min at 600g. Red blood cells were lysed by ACK lysis buffer. Collected tissues for OCT embedding were fixed in 4% PFA for 1h at 4°C and then incubated in 20% sucrose overnight. The samples were transferred into OCT filled cryomoulds and kept at -80°C. 10μm sections were cut with the cryostat and attached to coated Menzel Superfrost slides.

### Monocyte and macrophage subtype quantification by immunofluorescence

Slides were fixed in 4% PFA for 10mins at 4°C, incubated for 20mins at RT in blocking buffer (10% serum in PBS-Tween) and incubated with the primary antibody (diluted in PBS-Tween) over night at 4°C: Ly6G antibody for neutrophils (rat anti mouse Ly6G IgG2a, 0.5mg/ml, BD Pharmingen, 1:200), CD206 (rat anti-mouse CD206 IgG2a, 100μg/ml, Santa Cruz, 1:200), Gr1 (rat anti-mouse Gr1 IgG2b, 1mg/ml, Serotec, 1:500) and F4/80 (rat anti-mouse F4/80 IgG2b, 0.2mg/ml, Serotec, 1:100) to determine monocyte and macrophage subtypes. The slides were washed in PBS-Tween three times for 5mins and were then incubated with the secondary antibody (goat anti rat IgG Alexa Fluor 654, 2mg/ml, Invitrogen, 1:1000, diluted in PBS-Tween) for 2hrs at RT in the dark when needed. After washings, slides were stained with DAPI and coverslipped using mounting media. 60x photomicrographs were taken with confocal microscope (Nikon C1, Japan) and images analysed using the NIS-Elements BR 3.0 software.

### RNA Isolation, cDNA synthesis and Quantitative Real-time PCR analysis

RNA isolation was performed using QIAzol Lysis Reagent (Qiagen) and RNeasy Plus Universal kit (Qiagen) and cDNA was synthesized using SuperScript® III reverse transcriptase kit (Invitrogen), all according to the manufacturer’s instructions. To quantify the expression of M1 and M2 mRNA, real-time (RT)-PCR analysis was performed using SensiMix SYBR No-ROX Kit (Bioline) with specific primers for each gene (Listed in S1 Table). Relative expression differences were calculated using the comparative Ct (ΔCt) method using β-actin/ as a housekeeping gene.

### Statistics

All data represent mean ± SEM and all experiments were routinely performed in triplicate and represent ≥ 3 independent experiments. Results were analysed using nonparametric Mann-Whitney tests for 2-group comparisons and Kruskal-Wallis tests for ≥ 3 group comparisons, with subsequent pair-wise comparisons using Dunns test (GraphPad Prism version 4; Graph-Pad Software, Inc; San Diego, CA). *P*-values <0.05 were considered statistically significant.

## Results

### GW2580 treatment depleted peripheral blood Gr1^hi^ and F4/80^hi^ monocyte populations

**Fig 1:**
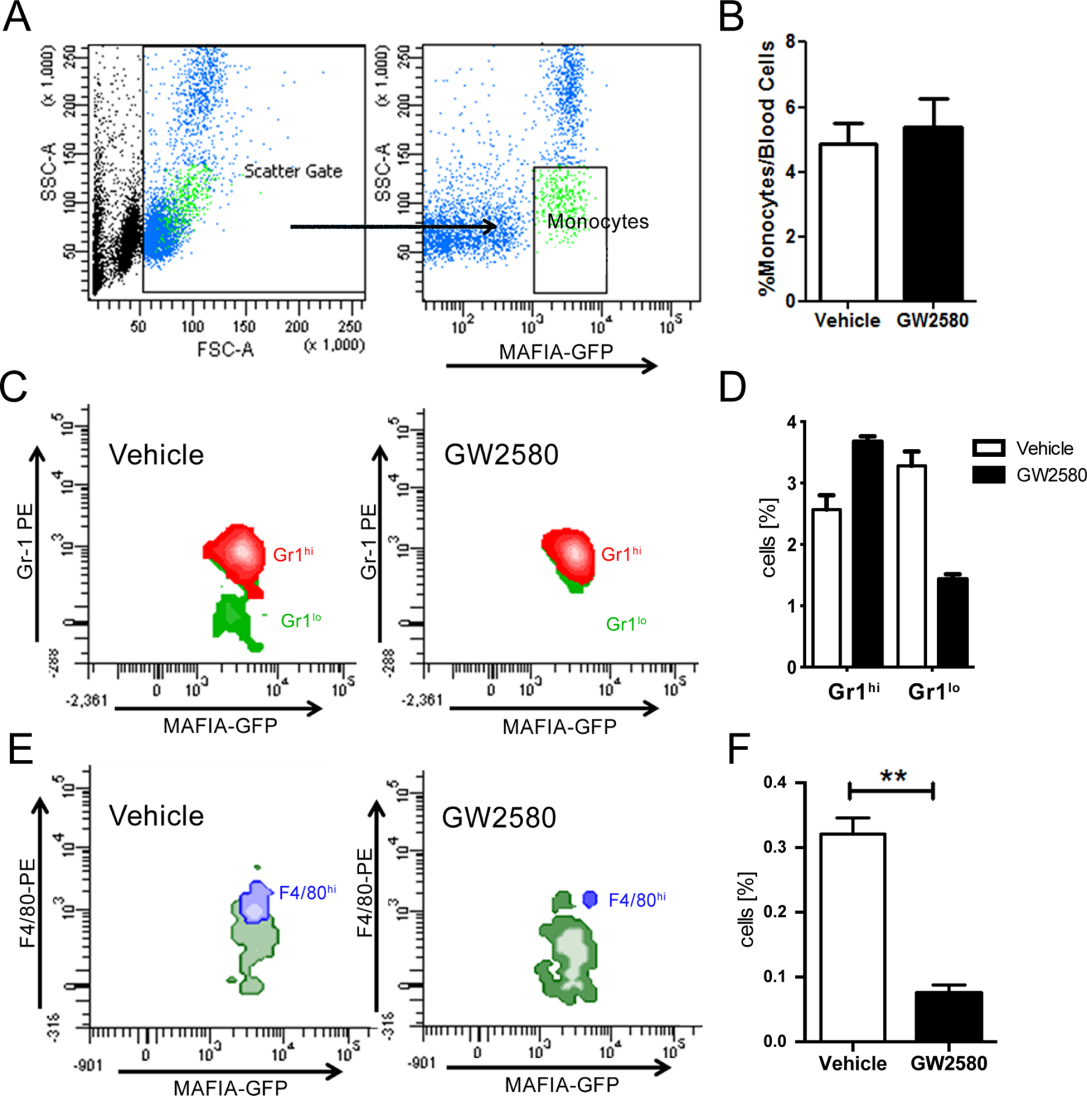
Depletion of the circulating monocyte and Gr1^lo^ and F4/80^hi^ populations following 1 week GW2580 treatment. A. Identification of total monocyte population in mouse blood using MAFIA-GFP. B Following one week of GW2580 treatment, no difference in total circulating monocytes was observed. FACS quantification of Gr1^hi^ (M1) and Gr1^lo^ (M2; C,D) and F4/80^hi^ (E,F) in total monocytes following 1 week GW2580 treatment (n = 6 and n = 4 animals per group, respectively).

To induce significant depletion of M2 macrophages, MAFIA transgenic mice were treated with GW2580 (160 mg/kg, daily) or Vehicle for one week orally. MAFIA mice expressing eGFP under the *Csf-1* receptor promoter control allowed tracking of CSF-1^+^ cells. There was no difference in GFP+ total monocytes in the circulation after one week of GW2580 treatment (Fig 1A and 1B). However, subtype analysis revealed that CSF-1R signaling inhibition depleted GFP+/Gr1^lo^ (**:p<0.001, Fig 1C and 1D) and GFP+/F4/80^hi^ monocytes in peripheral blood (**:p<0.001, Fig 1E and 1F). In contrast, the circulating Gr1^hi^ monocyte population was increased (**:p<0.001, Fig 1C and 1D). A similar reduction in Gr1^hi^ and F4/80^hi^ circulating monocyte populations has previously been observed by MacDonald *et al*. in response to CSF-1R blockade [12]. Parallel experiments using C57Bl6J mice (S2 Fig) demonstrated that Gr1^lo^ monocyte depletion was not related to the genetic background of the animals. These findings are in keeping with the evidence that CSF1R signaling inhibition by GW2580, reduces production of M-CSF-derived immunosuppressive monocytes while GM-CSF-derived monocytes remain unaffected in terms of viability, expression of differentiation markers and interleukin production [13].

### GW2580 treatment depleted the cardiac tissue resident M2 macrophage population

**Fig 2:**
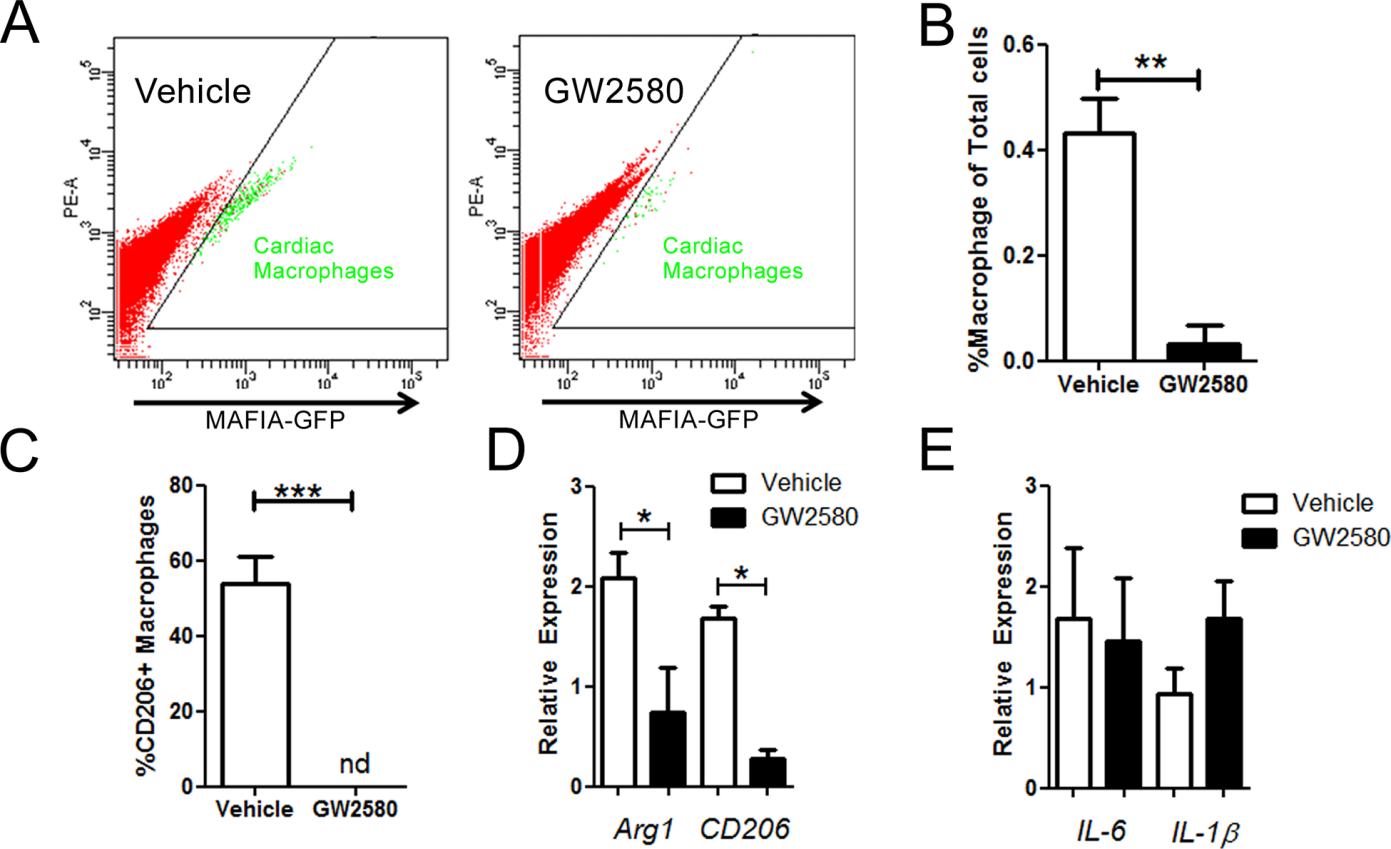
Depletion of the M2 cardiac macrophage populations following 1 week GW2580 treatment. A. Representative profiles of digested hearts from vehicle and GW2580-treated animals. B. Quantification of cardiac tissue-resident macrophages 1 week post-GW2580 treatment (n = 6 animals per group). C. Quantification of CD206^+^ M2 macrophages within the heart, 1 week post-GW2580 treatment (n = 6 animals per group, nd:not detected). D. mRNA expression relative to GAPDH of M2 markers Arg1 and CD206 and E. M1 markers IL6, IL1β, respectively, within the heart following 1 week GW2580 treatment (n = 4 animals per group). * *p*<0.05, ** *p*<0.01.

Interestingly, the GFP^+^ resident macrophage population, which are predominantly in an M2 polarity [1], was significantly decreased in the hearts of GW2580 treated mice compared to vehicle treated controls (**:p<0.001, Fig 2A and 2B). Consistent with findings in the circulation and spleen (S3 Fig), we also found that the number of M2 CD206^+^ macrophages was significantly reduced in the heart 1 week following treatment (Fig 2C). Furthermore, qRT-PCR experiments confirmed significant decreases in mRNA expression levels of M2 macrophage markers CD206 and Arg1 within the heart after 1 week GW2580 treatment (Fig 2D) whereas M1 markers Il6 and ILβ expression of expression remained unchanged (Fig 2E) indicating a potent and specific targeting of cardiac CD206^+^ M2 macrophages following CSF-1R signal inhibition. Increased apoptosis was also observed in the splenic M2/CD206+ population in the GW2580-treated animals compared to controls (S3 Fig).

### CD206^+^ M2 macrophage depletion significantly reduced ejection fraction 2 weeks post MI and increased infarct size.

**Fig 3:**
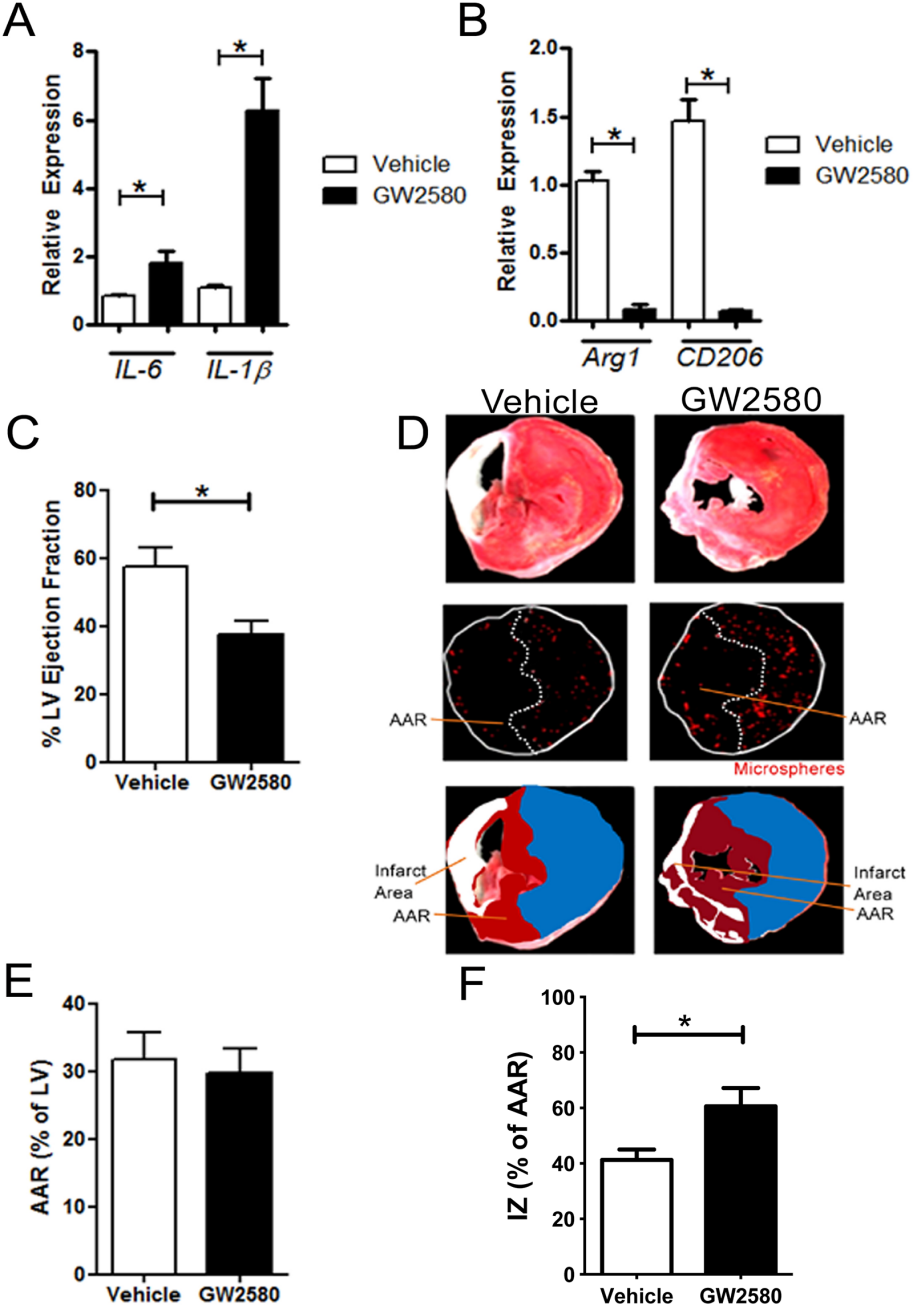
CD206+ M2 macrophage depletion reduces LV ejection fraction but not infarct size 2 weeks post MI in MAFIA mice. A. mRNA expression relative to GAPDH of M1 markers IL6, IL1B, and (b) M2 markers Arg1 and CD206 respectively within the heart 2 weeks post MI (n = 4–6 animals per group). C. Determination of ejection fraction based on pressure-volume loop measurements vehicle or GW2580-treated MAFIA mice 2 weeks post MI. (* *p*<0.05. n = 11–13 animals per group). D. Upper panels: representative MAFIA heart sections after TTC staining. Middle panels: representative MAFIA heart section under fluorescent microscope allowing the detection of coloured microspheres distributed in the perfused area. Lower panels: Infarct areas are represented as white, non-perfused area-at risk (AAR) of infarction (dark red) and perfused areas (blue) are highlighted. The area at risk corresponds to the non-perfused area. E. AAR/LV ratio quantification in MAFIA mice 2 weeks post MI using coloured microspheres. F. IZ/LV ratio quantification in MAFIA mice 2weeks post MI using TTC staining and coloured microspheres (* *p*<0.05. n = 11–13 animals per group).

M2 macrophages activated at a later stage, five to seven days post-MI in mice are considered to contribute to cardiac repair by establishing an anti-inflammatory environment favoring stable scar formation [3]. GW2580 treated mice hearts in the absence of infarction, did not show observable effects on cardiac homeostasis. GW2580 treated mice (with or without MI) did not show any significant differences in the heart weight /bodyweight ratio or in the lung weight/bodyweight ratio compared to vehicle treated mice. Furthermore cardiomyocyte size 2 weeks post MI in the remote zone did not differ between GW2580 and vehicle treated mice (S4 Fig). Following M2 macrophage depletion, MAFIA mice underwent MI surgery. Two weeks post-MI and after a total of three week treatment with the GW2580 drug, the inflammatory milieu of the heart shifted towards a more pro-inflammatory environment with M1 marker mRNA expression significantly increased (Fig 3A) and a concomitant reduction in M2 marker mRNA expression (Fig 3B). Functional assessment showed a 30% decrease in ejection fraction in the GW2580-treated group 2 weeks post-MI in comparison to the vehicle-treated group (Fig 3C) suggesting that M2 depletion had a deleterious effect on cardiac functional recovery post-MI. In addition to LVEF reduction, an increase in infarct size two weeks post-MI was observed (Fig 3D–3F). In a separate group of mice without MI, LVEF was determined as 55.5% ± 4.4 (p>0.05 vs. Vehicle group) suggesting no baseline effects of GW2580 on heart function.

### M2 macrophage depletion during MI was associated with decreased collagen deposition and sustained inflammatory cell infiltration within the infarct zone

**Fig 4:**
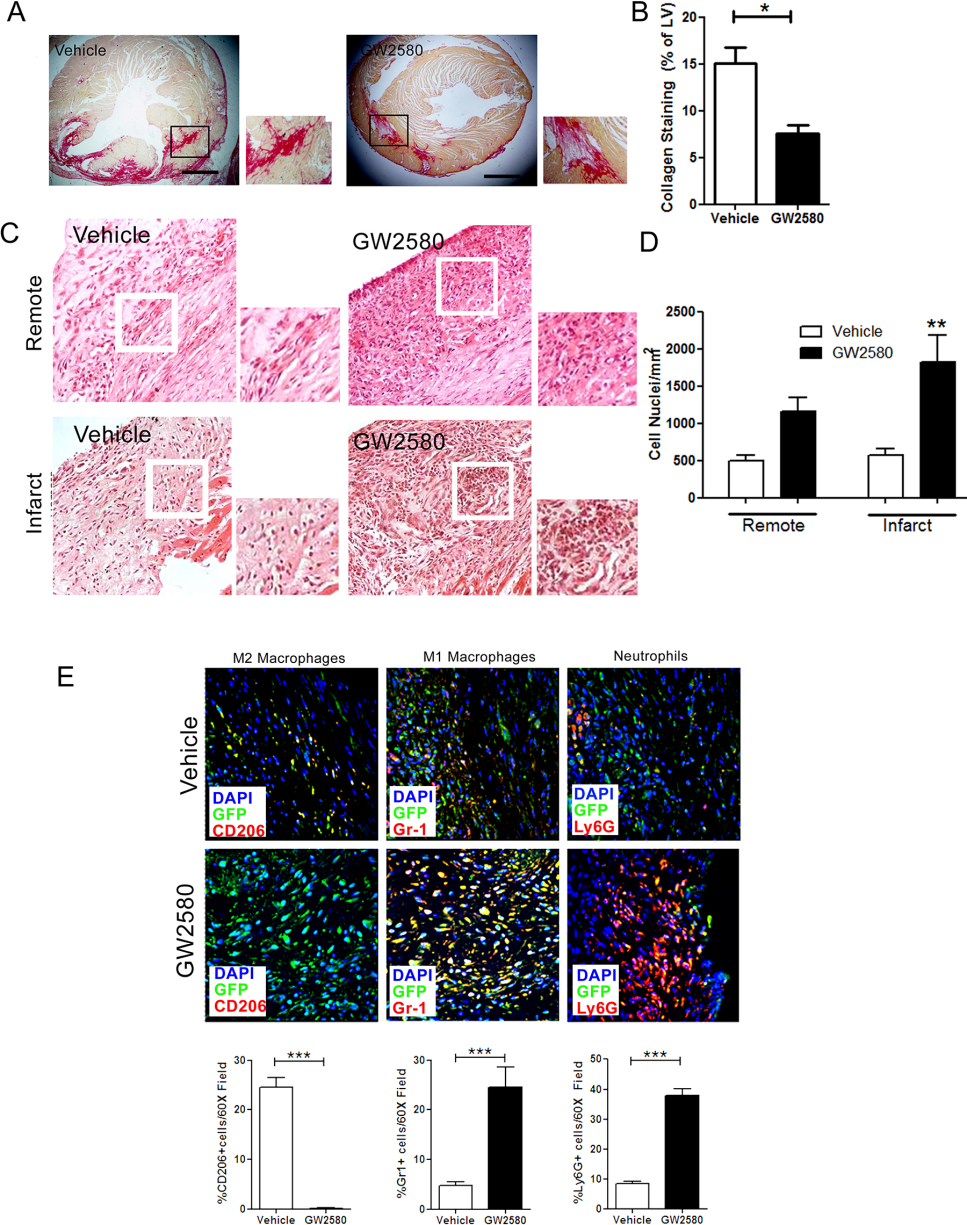
Effect of CD206+ M2 macrophage depletion on collagen deposition and cell infiltration within the infarct 2 weeks post MI in MAFIA mice. A. Representative Sirius Red staining on histological sections from MAFIA mice 2 weeks post MI. B. Quantification of collagen staining as a percentage of LV from MAFIA mice 2 weeks post MI (Scale bar: 1 mm, n = 6 animals per group, ≥8 images per animal). C. Representative hematoxilin and eosin staining on 20x infarct or remote histological section from MAFIA mice 2 weeks post MI showing an increase in inflammatory infiltrates in animals treated with GW2580. D. Quantification of nuclei number per mm^2^ in MAFIA remote and infarct zone in MAFIA mice 2 weeks post MI. (n = 4–6 animals per group). E. Representative images of immunofluorescent staining of CD206+ M2 macrophages, Gr1+ M1 macrophages and Ly6G+ neutrophils within the infarct zone from MAFIA mice 2 weeks post MI. Quantification of Ly6G+ neutrophil, Gr1+ M1 macrophage and CD206+ M2 macrophage infiltration within the infarct zone from MAFIA mice 2 weeks post MI (n = 4 animals per group, 10 images/animal). * *p*<0.05, ** *p*<0.01.

A comprehensive immunohistochemical analysis was performed to further characterize the cellular composition of the infarct area in terms of collagen deposition, cell infiltration and cell density. GW2580 treatment reduced collagen staining (**:p<0.001, Fig 4A and 4B) and increased cellular infiltration (**:p<0.001 Fig 4C and 4D) in the infarct zone. Multicolor confocal analysis revealed that consistent with the mRNA signals, infiltration of M2 macrophages defined as dual GFP and CD206 cells was decreased in the infarct zone, while both M1 macrophages (GFP+/Gr1+) and Ly6G+ neutrophils were increased (all ***:p<0.0001, Fig 4E). Sustained shift of the macrophage balance towards a pro-inflammatory phenotype at two weeks post MI thus was associated with impaired cardiac functional recovery in these animals.

## Discussion

In this study, the contribution of M2 cardiac tissue-resident macrophages in the response to experimental MI was investigated. Circulating monocyte Gr1^lo^ and F4/80^hi^ subpopulations and tissue resident M2 macrophages were selectively and significantly reduced following CSF-1R signaling inhibition. Post MI, M2 macrophage depletion resulted in a marked loss of left ventricular ejection fraction and infarct enlargement. In addition, depletion of M2 cardiac macrophages was associated with a reduction in collagen deposition and persistent infiltration of neutrophil and M1 macrophages in the infarct territory. These findings indicate that direct M2 depletion facilitates M1 persistence delaying the resolution of cardiac inflammation post MI.

Myocardial ischemia triggers a biphasic response from circulating cells; an initial pro-inflammatory stage featuring neutrophil and M1 monocyte/macrophage infiltration followed by recruitment of non-inflammatory M2 macrophages during the subsequent repair response [14,15]. Several lines of evidence, including reduction of M1-related gene expression through silencing of the IRF5 transcription factor [16] or blockade of CCR2 [17], both of which reduce cardiac injury, suggest that persistence of M1 macrophages is associated with a maladaptive repair response post MI. Moreover, studies of scavenger receptor class A (SR-A) deletion [18], and mineralocorticoid receptor activation [19] have reported an association between an M2 to M1 macrophage polarity shift and exacerbation of cardiac inflammation, fibrosis and ventricular dysfunction. Taken together, these studies hint at the existence of an optimal phenotypic balance between M1 and M2 cardiac macrophages in facilitating a complete cardiac repair response.

The origins and maintenance of the complement of cardiac tissue resident macrophages has been an area of active investigation over recent years. Utilizing various fate mapping methodologies, a concept of an embryonic origin for tissue resident macrophages which are maintained in the steady state by local proliferation has emerged [20]. However, in the event of macrophage depletion following cardiac injury, circulating monocytes may replenish this tissue pool at least in the acute phase before local proliferation resumes [21]. Here we demonstrate that inhibition of CSF-1R signalling results in depletion of both circulating anti-inflammatory monocytes and tissue resident macrophages, which are associated with sustained, elevated pro-inflammatory cell infiltration in the infarcted heart. Evidence from MacDonald and colleagues [12] in addition to the current study have suggested an association between the presence of Gr1^lo^ and F4/80^hi^ circulating monocyte populations and that of tissue resident macrophages across multiple organs. CSF-1 signalling is vital to the maintenance of both steady state tissue resident macrophages and their replenishment following infarction from putative extra-cardiac sources.

To date, no study has assessed specific disruption of resident M2 macrophages within the heart to more clearly define their contribution to the resolution of myocardial injury post-ischemia. Collectively, our data indicate that depletion of M2 tissue resident macrophages results in a preponderance of M1 macrophage phenotype that is temporally prolonged and associated with increased inflammatory cell infiltration, impaired scar formation, infarct expansion and loss of LV contractile function. A similar outcome has been previously described in MMP28 deficient mice, in which indirect inhibition M2 macrophage activation, also led to cardiac dysfunction [22]. MMP up-regulation follows the necrotic loss of cardiomyocytes post MI degrading interstitial collagen structures and disrupting the integrity of the coronary microvasculature [23]. This may trigger an M2 macrophage-driven pro-fibrotic response thought to limit further tissue damage and loss of parenchymal cell volume [24]. The relative spatial and temporal composition of M1 and M2 macrophages within the heart post MI may thus determine the level of scar formation, parenchymal cell loss and structure/function recovery as much as the initial infarct size.

Total depletion of monocytes/macrophage by clodronate-encapsulated in liposomes results in impaired wound healing post MI [25] but this depletion method, relying on the phagocytic activity of all monocytes and macrophages does not differentiate between pro- or anti-inflammatory phenotypic polarity exhibited by phagocytic cells, nor the contribution of either circulating or tissue resident macrophages. Our M2 macrophage depletion experiments, suggest that disproportionate activation of M1 macrophages or lack of countervailing M2 phenotype shift may potentiate tissue damage exacerbating maladaptive MI remodeling.

One limitation of our study involves the relatively small infarct generation strategy which resulted in preservation of global LVEF(~58%) in the vehicle treated group. A larger infarct however would have resulted in increased incidence of rupture-related mortality as well as difficulties in the assessment cardiac repair mechanisms following macrophage depletion. Although results of clinical trials targeting the overall inflammatory response post MI have to date been disappointing [26], we posit that collateral targeting of the M2 population in this context may impair cardiac functional recovery. Promotion of the M2 macrophage polarity may be enhanced by delivery of colony stimulating factor-1 (CSF-1), the major ligand for M2 transition, into the infarct zone [6]. In a rat model, parenteral delivery of recombinant protein, or more effectively, CSF-1 cDNA overexpressed in myoblasts facilitated cardiac repair in the heart post I/R [6]. Sustained CSF-1 delivery was associated with additional benefits to infarct size, scar formation and left ventricular function [6]. Clinical realization of CSF-1 or its analogues as a therapeutic in cardioprotection may however require a more sophisticated delivery approach, localized to the area of infarction and sustained throughout the recovery phase. Moreover, a more nuanced modulation of M1 versus M2 macrophage polarization may lead to a more effective therapeutic strategy promoting cardiac repair by augmenting endogenous healing mechanisms.

## Supporting Information

S1 Fig Staining controls for flow cytometry experiments. Isotype controls and Gr1 staining alone done in C57/Bl6 mice in additon to IgG-PE staining in MAFIA mice showing application of correct spectral overlap and PMT voltage settings.
(PDF)

S2 Fig Depletion and increased apoptosis of splenic M2 macrophages following GW2580 treatment. A Overall splenic macrophage population in the spleen detected by MAFIA-GFP was reduced by GW2580 treatment compared with Vehicle (*:p<0.05). B From the total MAFIA-GFP splenic macrophage population, GW2580 increased the proportion of M1 while decreasing the M2 macrophages in the spleen (***:p<0.0001, **:p<0.001). C: GW2580 treatment increases cell death of overall splenic macrophages (*:p<0.05). D In the CD206+ M2 splenic macrophage subset, GW2580 treatment increases cell death as detected by 7-AAD (*: p<0.05). C: Apoptosis of in situ splenic macrophage verified by in situ TUNEL staining (red) of MAFIA-GFP (green) splenic cells. Nuclei were counterstained with DAPI.
(PDF)

S3 Fig M2 macrophage depletion in C57/Bl/6 mice. A,B. FACS quantification of Gr1hi (M1) and Gr1lo (M2) in total monocytes from wild type C57/Bl6 mice following 1 week GW2580 treatment (n = 5 animals per group, respectively). C,D Immunofluorescence analysis of CD206+ cells (red) in heart tissue following 1 week vehicle or GW2580 treatment (Scale bar: 10 μm, **:p>0.001, nd: not detected).
(PDF)

S4 Fig M2 macrophage depletion in C57/Bl/6 mice. A, Survival rates of Vehicle or GW2580-treated mice post MI B.Ratio of heart and lung weight in mg to body weight in grammes showing no alterations in heart size resulting from GW2580 treatment. C Measurement of serum troponin 24 hours post MI showing no differences in initial infarct generation. D Area-at-Risk measurement using infusion of two distinct coloured microsphere suspensions showing equivalent AAR territories at the time of ligation and two weeks later. E No increase in cardiomocyte size as a measure of hypertrophy was observed.
(PDF)

S1 Table Primers used for qRTPCR. (PDF)
