# Supplementary material for: Systemic and Cardiac Depletion of M2 Macrophage through CSF-1R Signaling Inhibition Alters Cardiac Function Post Myocardial Infarction
Source: PLoS One. 2015 Sep 25;10(9):e0137515. doi: 10.1371/journal.pone.0137515 (PMC4583226; doi:10.1371/journal.pone.0137515)
Supplement: S1 Fig — Isotype controls and Gr1 staining alone done in C57/Bl6 mice in additon to IgG-PE staining in MAFIA mice showing application of correct spectral overlap and PMT voltage settings. (PDF) [file pone.0137515.s001.pdf]

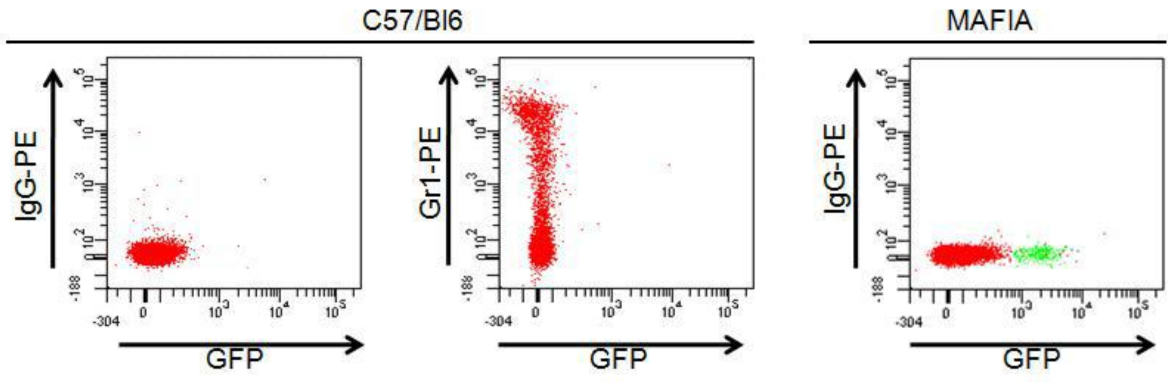

Supplementary Figure 1 Staining controls for flow cytometry experiments
Isotype controls and Gr1 staining alone done in C57/Bl6 mice in additon to IgG-PE staining in MAFIA mice showing application of correct spectral overlap and PMT voltage settings
