# Supplementary material for: Systemic and Cardiac Depletion of M2 Macrophage through CSF-1R Signaling Inhibition Alters Cardiac Function Post Myocardial Infarction
Source: PLoS One. 2015 Sep 25;10(9):e0137515. doi: 10.1371/journal.pone.0137515 (PMC4583226; doi:10.1371/journal.pone.0137515)
Supplement: S2 Fig — A Overall splenic macrophage population in the spleen detected by MAFIA-GFP was reduced by GW2580 treatment compared with Vehicle (*:p<0.05). B From the total MAFIA-GFP splenic macrophage population, GW2580 increased the proportion of M1 while decreasing the M2 macrophages in the spleen (***:p<0.0001, **:p<0.001). C: GW2580 treatment increases cell death of overall splenic macrophages (*:p<0.05). D In the CD206+ M2 splenic macrophage subset, GW2580 treatment increases cell death as detected by 7-AAD (*: p<0.05). C: Apoptosis of in situ splenic macrophage verified by in situ TUNEL staining (red) of MAFIA-GFP (green) splenic cells. Nuclei were counterstained with DAPI. (PDF) [file pone.0137515.s002.pdf]

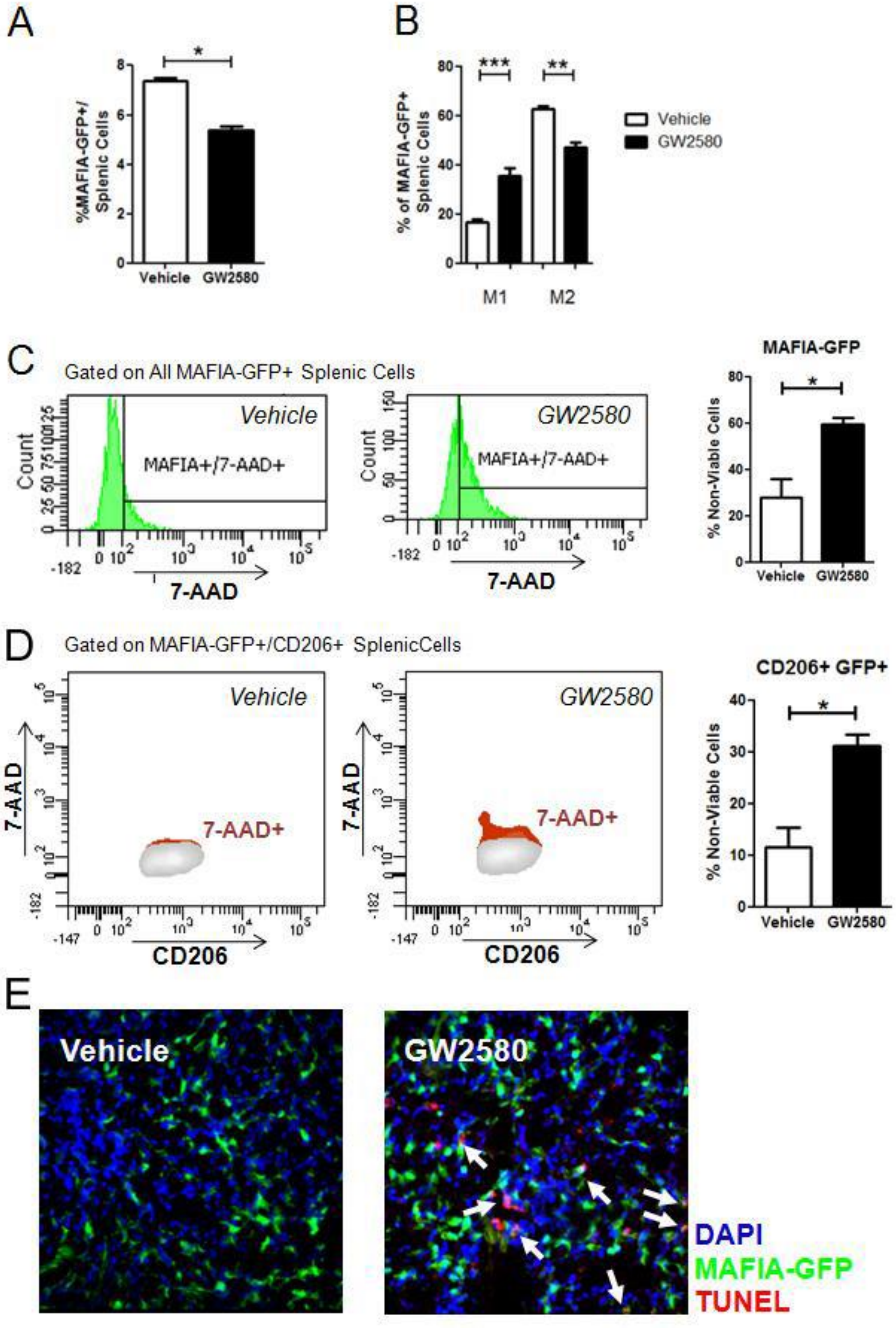

Supplementary Figure 2 Depletion and increased apoptosis of splenic M2 macrophages following GW2580 treatment. A Overall splenic macrophage population in the spleen detected by MAFIA-GFP was reduced by GW2580 treatment compared with Vehicle (*:p<0.05). B From the total MAFIA-GFP splenic macrophage population, GW2580 increased the proportion of M1 while decreasing the M2 macrophages in the spleen (***:p<0.0001, **:p<0.001). C: GW2580 treatment increases cell death of overall splenic macrophages (*:p<0.05). D In the CD206+ M2 splenic macrophage subset, GW2580 treatment increases cell death as detected by 7-AAD (*: p<0.05). C: Apoptosis of *in situ* splenic macrophage verified by *in situ* TUNEL staining (red) of MAFIA-GFP (green) splenic cells. Nuclei were counterstained with DAPI.
