# Supplementary material for: Systemic and Cardiac Depletion of M2 Macrophage through CSF-1R Signaling Inhibition Alters Cardiac Function Post Myocardial Infarction
Source: PLoS One. 2015 Sep 25;10(9):e0137515. doi: 10.1371/journal.pone.0137515 (PMC4583226; doi:10.1371/journal.pone.0137515)
Supplement: S3 Fig — A,B. FACS quantification of Gr1hi (M1) and Gr1lo (M2) in total monocytes from wild type C57/Bl6 mice following 1 week GW2580 treatment (n = 5 animals per group, respectively). C,D Immunofluorescence analysis of CD206+ cells (red) in heart tissue following 1 week vehicle or GW2580 treatment (Scale bar: 10 μm, **:p>0.001, nd: not detected). (PDF) [file pone.0137515.s003.pdf]

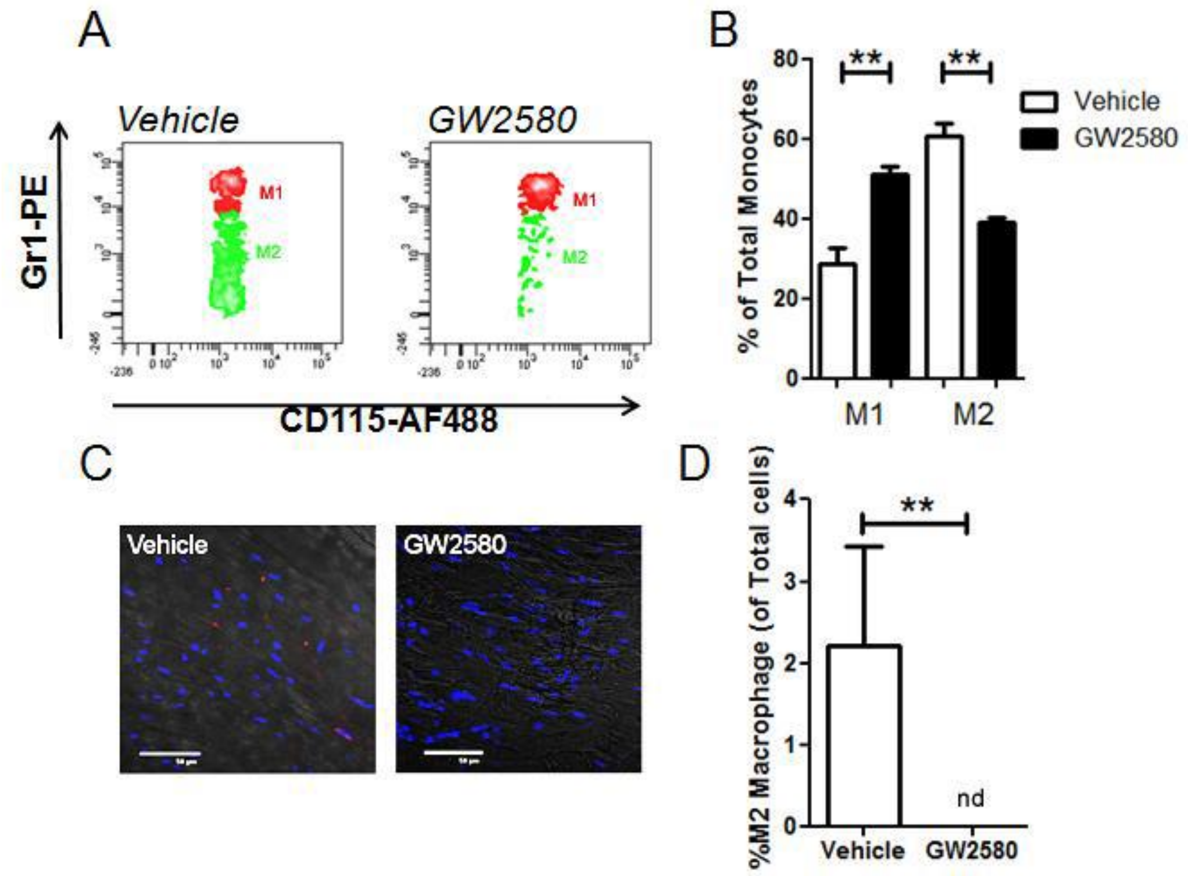

Supplementary Figure 3 M2 macrophage depletion in C57/Bl/6 mice.
A,B. FACS quantification of $Gr1_{hi}$ (M1) and $Gr1_{lo}$ (M2) in total monocytes from wild type C57/Bl6 mice following 1 week GW2580 treatment (n = 5 animals per group, respectively). C,D Immunofluorescence analysis of CD206+ cells (red) in heart tissue following 1 week vehicle or GW2580 treatment (Scale bar: 10 µm, **:p>0.001, nd: not detected).
