# Supplementary material for: Systemic and Cardiac Depletion of M2 Macrophage through CSF-1R Signaling Inhibition Alters Cardiac Function Post Myocardial Infarction
Source: PLoS One. 2015 Sep 25;10(9):e0137515. doi: 10.1371/journal.pone.0137515 (PMC4583226; doi:10.1371/journal.pone.0137515)
Supplement: S4 Fig — A, Survival rates of Vehicle or GW2580-treated mice post MI B.Ratio of heart and lung weight in mg to body weight in grammes showing no alterations in heart size resulting from GW2580 treatment. C Measurement of serum troponin 24 hours post MI showing no differences in initial infarct generation. D Area-at-Risk measurement using infusion of two distinct coloured microsphere suspensions showing equivalent AAR territories at the time of ligation and two weeks later. E No increase in cardiomocyte size as a measure of hypertrophy was observed. (PDF) [file pone.0137515.s004.pdf]

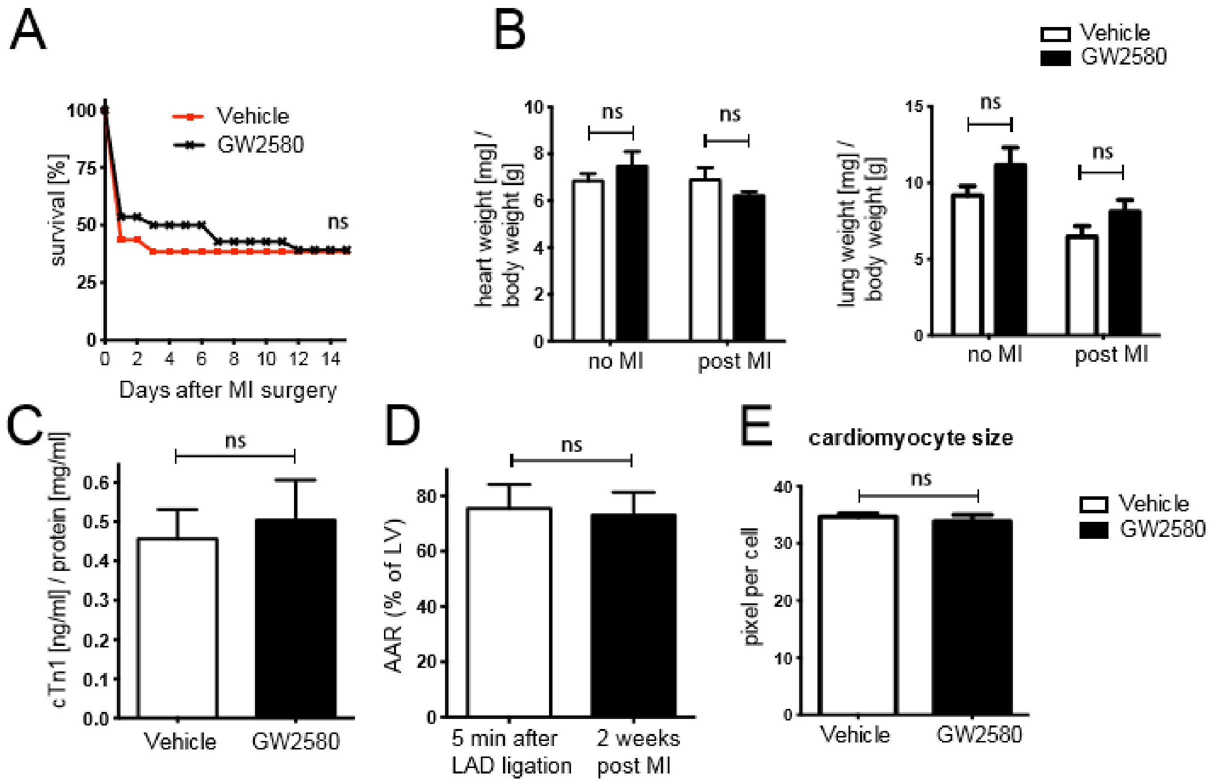

Supplementary Figure 4 M2 macrophage depletion in C57/Bl/6 mice.
A, Survival rates of Vehicle or GW2580-treated mice post MI B.Ratio of heart and lung weight in mg to body weight in grammes showing no alterations in heart size resulting from GW2580 treatment. C Measurement of serum troponin 24 hours post MI showing no differences in initial infarct generation. D Area-at-Risk measurement using infusion of two distinct coloured microsphere suspensions showing equivalent AAR territories at the time of ligation and two weeks later. E No increase in cardiomocyte size as a measure of hypertrophy was observed.
