# Supplementary material for: Systemic and Cardiac Depletion of M2 Macrophage through CSF-1R Signaling Inhibition Alters Cardiac Function Post Myocardial Infarction
Source: PLoS One. 2015 Sep 25;10(9):e0137515. doi: 10.1371/journal.pone.0137515 (PMC4583226; doi:10.1371/journal.pone.0137515)
Supplement: S1 Table — (PDF) [file pone.0137515.s005.pdf]

Supplementary Table 1 Primers used for qRTPCR

**Gene**	**Forward**	**Reverse**
IL6	ccagttgccttcttgggact	ggtctgttgggagtggtatcc
IL1beta	Ctggtgtgtgacgttcccatta	ccgacagcacgaccgttt
ARG1	aggaactggctgaagtggtta	gatgagaaaggaaagtggctgt
CD206	caggtgtgggctcaggtagt	tgtggtgagctgaaaggtga
